# Clinical efficacy analysis of paxlovid in children with hematological diseases infected with the omicron SARS-CoV-2 new variant

**DOI:** 10.3389/fped.2023.1160929

**Published:** 2023-04-25

**Authors:** Yixian Li, Yong Liu, Luping Wen, Hui Chen, Wenqing Wang, Mengyao Tian, Yucai Cheng, Hongman Xue, Chun Chen

**Affiliations:** ^1^ Pediatric Hematology Laboratory, Division of Hematology/Oncology, Department of Pediatrics, The Seventh Affiliated Hospital of Sun Yat-Sen University, Shenzhen, China; ^2^ Department of Pharmacy, The Seventh Affiliated Hospital of Sun Yat-Sen University, Shenzhen, China; ^3^ Research Center, The Seventh Affiliated Hospital of Sun Yat-sen University, Shenzhen, China

**Keywords:** clinical efficacy, PAXLOVID, novel coronavirus, hematologic diseases, children

## Abstract

**Objective:**

To summarize the clinical characteristics of children with hematological malignancies co-infected with novel coronavirus and explore the safety and effectiveness of Paxlovid treatment.

**Methods:**

From December 10, 2022, to January 20, 2023, the clinical data of children with hematological diseases diagnosed with novel coronavirus infection in the outpatient and emergency department of the Seventh Affiliated Hospital of Sun Yat-sen University were retrospectively analyzed.

**Results:**

According to whether to give paxlovid or not, it is divided into group A (paxlovid group) and group B (non-paxlovid group). The length of fever was 1–6 days in group A and 0–3 days in group B. The viral clearance time was shorter in group A than in group B. The inflammatory indexes CRP and PCT were significantly higher in group A than in group B (*P *< 0.05). Twenty patients were followed up for 1 month after leaving the hospital, and there were 5 cases of reappearance of fever, 1 case of increased sleep, 1 case of physical fatigue and 1 case of loss of appetite within 2 weeks.

**Conclusions:**

Paxlovid has no apparent adverse reactions in children 12 years old and younger with underlying hematological diseases infected with the new coronavirus. Focusing on the interaction between paxlovid and other drugs is necessary during the treatment.

## Introduction

In 2019, severe acute respiratory syndrome coronavirus 2 (SARS-CoV-2) began to spread worldwide. SARS-CoV-2 has undergone several significant mutations in the past three years from Alpha to Omicron, and the 2019 novel coronavirus disease (COVID-19) epidemic continues to spread worldwide (1). The Omicron variant, the primary circulating strain in the world, has stronger intrinsic infectivity through binding to human ACE2 (angiotensin-converting enzyme 2) and the rapid replication ability of the virus itself (2, 3). At the same time, it has a robust immune escape ability to avoid the immune response of human vaccination or infection, both of which make it more adaptable (3). According to studies, SARS-CoV-2 is mainly transmitted through respiratory droplets and close contact and can also be transmitted through aerosols and contact with virus-contaminated surfaces, and the population is generally susceptible (4).

With the opening of China's anti-epidemic policy (the new ten measures) on December 7, 2022, the number of domestic infections has increased dramatically. However, due to the cancellation of nucleic acid testing, there is no relevant statistical data on the number of infections. According to a study published by the US Centers for Disease Control and Prevention (CDC) Weekly report, during the Omicron epidemic in the United States from April to August 2022, the hospitalization rate of COVID-19 patients was 0.75%, and the mortality rate was 0.03% (5). Children with underlying diseases and low immune function are at risk of severe disease, and safe and effective specific antiviral drugs are urgently needed. Clinical data from the phase 2/3 double-blind, randomized controlled trial (EPIC-HR) showed that Paxlovid treatment in the early stage of COVID-19 disease could reduce the risk of disease progression to severe disease and rapidly reduce SARS-CoV-2 viral load without obvious safety concerns (6). Paxlovid is packaged with a combination of Nirmatrelvir and Ritonavir. Nematavir is a major protease (Mpro) inhibitor of SARS-CoV-2, which can prevent viral replication (7, 8). Ritonavir, an HIV protease inhibitor with low antiviral activity, has no antiviral activity against SARS-CoV-2 but has vigorous CYP3A4 inhibition activity, which can delay the metabolism of nematavir in the liver and the duration of retention in the body (7, 9). Paxlovid was approved by the US Food and Drug Administration (FDA) on December 22, 2021, for use in adult and pediatric patients (12 years and older, body weight ≥40 kg) with mild-to-moderate COVID-19 who tested positive for SARS-CoV-2 virus and had high-risk factors for progression to severe disease (10). Due to the immature organ function in children, the pharmacokinetics of Paxlovid in children may differ from that in adults. The interaction with other drugs can lead to various adverse reactions (11). Therefore, its efficacy and safety in children with hematological diseases must be further studied.

This is the first study to investigate the clinical characteristics of COVID-19 infection and the safety and efficacy of Paxlovid treatment in children with hematological diseases by analyzing the clinical data of 20 patients with hematological diseases in the Seventh Affiliated Hospital of Sun Yat-sen University. It can provide pediatricians with decision-making help in the clinical diagnosis and treatment of children with hematological malignancies infected with SARS-CoV-2 virus, thereby further reducing the incidence of severe infection and mortality in children.

## Patients and methods

### Patients

Data were collected from 20 children with underlying diseases admitted to the Seventh Affiliated Hospital of Sun Yat-sen University from December 10 to January 20, 2022. They were diagnosed with SARS-CoV-2 infection by reverse transcription polymerase chain reaction (RT-PCR) detection of oropharyngeal swabs and quantitative SARS-CoV-2 viral load. The diagnostic criteria for novel coronavirus infection following the 2019 Guidelines for the Treatment of Coronavirus Disease 2019 (COVID-19) issued by the National Institutes of Health of the United States (12) and the “Diagnosis and Treatment of Novel coronavirus Pneumonia (Trial 10th Edition)” issued by the Health Commission of China (13). According to NCCN Guidelines for the Prevention and Treatment of Cancer-Associated Infections, Version 2022.3 (14), the severity of COVID-19 infection in the 20 children was not severe (mild or moderate).

20 children with mild symptoms were divided into two groups (group A and group B), according to the high-risk factors for children with severe and critical illness in the “Diagnosis and Treatment Plan for Novel Coronavirus Pneumonia (Trial Version 10)” issued by the National Health Commission of my country. Group A: the group of children with hematologic neoplastic diseases treated with paxlovid(case1–9), in which there are two high-risk factors for the development of severe and critical illnesses, including case 4 with combined hematologic neoplastic diseases and pulmonary imaging suggestive of neocoronary pneumonia, with the possibility of developing severe and critical illnesses, and the remaining cases in the group are children with hematologic diseases and combined granulocyte deficiency. Group B: patients with hematological malignancies who did not receive paxlovid treatment (case10–20) had only a single risk factor for hematological malignancies and no other risk factors for severe or critical illness. The 9 paxlovid treatment cases obtained the informed consent of their legal guardians before administration. This study was approved by the Ethics Committee of the Seventh Hospital of Sun Yat-sen University (Grant No. YQ-C-2023–16–01).

### Research methods

Clinical data of children with hematological diseases diagnosed with the 2019-ncov infection in the outpatient and emergency department of the Seventh Affiliated Hospital of Sun Yat-sen University from December 10 to January 20, 2022, were retrospectively collected. The time to viral clearance was defined as the time from the first positive nucleic acid test or the onset of symptoms (whichever was earlier) to the first negative nucleic acid test (two consecutive tests).

### Statistical

SPSS 26.0 software was used for statistical analysis, and descriptive statistical analysis was used. The average distribution measurement data were expressed as Mean ± standard deviation (Mean ± SD), the variables between the two groups were compared by *t*-test, and the count data were expressed as the number of cases or percentage. When comparing categorical variables between groups, the Chi-square test was used when the sample size was >40; Fisher's exact probability test was used when the sample size was ≤40. *P *< 0.05 was considered statistically significant (15).

## Results

### Characteristics of the patients

20 children with hematological diseases infected with 2019-nCoV were included in this study Among them, children with leukemia accounted for 75% (6 cases in group A and 9 cases in group B), children with aplastic anemia accounted for 20% (2 cases in group A and 2 cases in group B), children after hematopoietic stem cell transplantation accounted for 5% (1 case in group A);

There were 9 cases in group A, 7 males and 2 females, aged 4–14 years, mean (7.67 ± 3.162) years, weight range 18–60 kg; 11 cases in group B, 7 males and 4 females, aged 2–11 years, mean (5.73 ± 3.165) years, weight range 10.5–32 kg. The differences in gender, age, weight and type of underlying disease between the two groups were not statistically (*P *> 0.05).

### Paxlovid treatment group

There were 2 children (22.2%) who received the novel coronavirus vaccine, 9 children (100%) had a fever, 6children 66.7%) had respiratory symptoms,4 children (44.4%) had gastrointestinal symptoms, and 2 children (22.2%) had nervous system symptoms, but no skin and mucosa damage, muscle pain, conjunctivitis, etc. (shown in Table 1). There were 8 cases of leucopenia (88.9%, cases 1, 2, 3, 5, 6, 7, 8, 9) in the initial stage of infection, 8 cases of neutropenia (83.3%, cases 1, 2, 3, 5, 6, 7, 8, 9), and 8 cases of anemia (88.9%), thrombocytopenia in 5 cases (55.6%), CRP elevation in 6 cases (66.7%), and PCT elevation in 6 cases (66.7%). There was 1 case (11.1%) of elevated liver enzymes, 0 case of elevated creatinine, 0 cases of abnormal cardiac enzymes, 0 cases of abnormal coagulation function, and 1 case of pneumonia revealed by lung CT examination (shown in Table 2). The 9 children in group A were all treated with paxlovid after consulting with the Department of Infectious Diseases and the Department of Pharmacy and obtaining the signed consent of their family members. Each dose was adult dose × body weight/40 kg, and the frequency of administration was less than 20 kg once a day and more than 20 kg twice a day for 5 days. CRP and (or) PCT increased in 9 children in group A, suggesting the possibility of secondary bacterial infection. According to clinical symptoms and “Guidelines for Clinical Application of Antibacterial Drugs in Patients with Neutropenia and Fever in China (2020 Edition)” diagnosis and treatment recommendations (16), all patients were treated with antibiotics. Six children in group A (cases 1, 2, 3, 5, 7, 9, ANCA < 1.0 × 10^9^/l) were treated with granulocyte colony-stimulating factor or gamma globulin for immune support. There were 3 children in group A (cases 1, 3, and 5) with drug interactions, including cyclosporine, prednisone, and dasatinib. After clinical evaluation of the severity of treatment indications for primary blood diseases. In case 1, the dose of cyclosporine was adjusted, and the dose was reduced after hypertension occurred; in case 3, the dose of dasatinib was reduced by 75%; in case 5, cyclosporine was suspended, and specialized treatment was given after the new coronavirus infection was cured. The average length of fever was 1–6 days (mean 2.89 ± 1.965 days), and clinical symptoms lasted up to 1 month, virus clearance took 5–15 days. There were 2 cases of fever again 3 days after the body temperature improved (in case 3, the concurrent bacterial infection was not completely cured, and in case 5, secondary sepsis was considered). Adverse reactions during paxlovid treatment were monitored: there were 2 cases (22.2%) with bitter taste and 1 case (11.1%) with diarrhea after administration, and no case showed symptoms of elevated liver enzymes and creatinine. (shown in Table 3).

**Table 1:** Clinical manifestations of 9 cases of children with hematological diseases in group A infected with novel coronavirus.

Patients	1	2	3	4	5	6	7	8	9
Gender	Male	Male	Male	Male	Male	Male	Female	Male	Female
Age(years)	4	10	14	4	6	8	9	8	6
Weight (kg)	18	31	60	21	19	30	31	29	20
Basic Illness	AA, after hematopoietic stem cell transplantation	B-ALL (relapsed, TEL/AML1+), myelosuppressive period after chemotherapy, drug-induced liver damage, G6PD deficiency	CML (blast change phase, BCR/ABL1 P210 positive), myelosuppressive phase after chemotherapy	ALL (B, IR, CR)	AA (trilineage), G6PD deficiency	AA (erythroid and megakaryoline), alpha thalassemia	ALL (B, IR)	ALL (B, IR)	ALL (B, HR)
COVID-19 Vaccination Status	0	0	2	0	2	0	0	0	0
Fever	Yes, 37.4°C	Yes, 38.6°C	Yes, 37.5°C	Yes, 39.5°C	Yes, 39.8°C	Yes, 39°C	Yes, 38.9°C	Yes, 39.4°C	Yes, 39°C
Duration of Fever	1 day	2 days	1 day	6 days	5 days	5 days	2 days	3 days	1 day
Cough/Stuffy nose/Runny nose/Sore throat	Yes	No	No	Yes	No	cough	cough	cough	cough
Rash/Mouth sores	No	No	No	No	No	No	No	No	No
Conjunctivitis	No	No	No	No	No	No	No	No	No
Headache/Dizziness	No	No	No	Yes	No	Yes	No	No	No
Muscle Pain	No	No	No	No	No	No	No	No	No
Vomiting/Diarrhea/Stomachache	No	No	No	No	Stomachache	No	Vomiting	No	No

**AA**, aplastic anemia; **ALL**, acute lymphoblastic leukemia; **CML**, chronic myelogenous leukemia.

**Table 2:** Laboratory test results of 9 children with hematological diseases in group A infected with novel coronavirus.

Patients	1	2	3	4	5	6	7	8	9
Clinical classification of new coronavirus infection.	Light	Light	Light	Medium	Light	Light	Light	Light	Light
Chest imaging	NS	NS	NS	Multiple patchy shadows and ground-glass shadows in both lungs	–	–	–	–	–
New coronavirus nucleic acid CT value	ORF/N 26.12/26.62	ORF/N 29.03/29.59	ORF/N 32.50/32.06	ORF/N 33.07/33.59	ORF/N 22.71/23.49	ORF/N 33.17/33.28	ORF/N 27.54/27.23	ORF/N 25.64/26.76	ORF/N 28.30/28.12
WBC(×10^9^/L)	1.57↓	1.33↓	0.93↓	5.18	2.18↓	2.52↓	1.15↓	2.07↓	0.89↓
ANCA( × 10^9^/L)	0.22↓	0.76↓	0.06↓	3.28	0.20↓	1.23↓	0.16	0.10↓	0.02↓
LC( × 10^9^/L)	1.11↓	0.18↓	0.87↓	1.43	1.93	1.06↓	0.98	1.95	0.81↓
Hb(g/L)	89↓	75↓	85↓	117	81↓	56↓	87↓	90↓	59↓
PLT( × 10^9^/L)	5↓	255	10↓	159	59↓	9↓	105	210	61↓
CRP (mg/L)	12.75↑	18.33↑	8.51	5.81	18.00↑	23.96↑	26.12↑	9.66	17.30↑
PCT (ng/ml)	0.20↑	0.84↑	0.05	<0.05	0.59↑	0.73↑	0.95↑	<0.05	0.56↑
ALT(U/L)	24	81↑	18	13	12	17	22	31.05	25
AST(U/L)	28.06	80.89↑	14	26.17	28.85	29.95	28.06	33	27

**WBC**, white blood cell count; **ANCA**, neutrophil count; **LC**, lymphocyte count; **Hb**, hemoglobin; **PLT**, platelet count; **CRP**, C-reactive protein; **PCT**, procalcitonin; **ALT**, alanine aminotransferase; **AST**, aspartate aminotransferase; **NS**, not detected; —: The result is normal.

**Table 3:** Treatment of 9 children with hematological diseases in group A who were infected with novel coronavirus.

Patients	1	2	3	4	5	6	7	8	9
Drugs to treat COVID-19	Naimatevir 150 mg/Ritonavir 50 mg q12 h	Naimatevir 225 mg/Ritonavir 75 mg q12 h	Naimatevir 300 mg/Ritonavir 100 mg q12 h	Naimatevir 150 mg/Ritonavir 50 mg q12 h	Naimatevir 150 mg/Ritonavir 50 mg q12 h	Naimatevir 150 mg/Ritonavir 50 mg q12 h	Nematavir 225 mg/ritonavir 75 mg q12 h	Nematavir 150 mg/ritonavir 50 mg q12 h	Nematavir 150 mg/ritonavir 50 mg q12 h
Interactions between drugs	Cyclosporine (reduced), prednisone (unchanged)	No	Dasatinib (100 mg qd changed to 50 mg qod)	No	Cyclosporine (discontinued)	No	No	No	No
Oxygen therapy	No	No	No	No	No	No	No	No	No
Combination antibiotics	Cefixime	Cefoperazone	Piperacillin	Cefoperazone	Cefoperazone	Cefuroxime	Cefoperazone	Piperacillin	Cefoperazone
Gamma Globulin	200-400 mg/kg/d × 5d	160 mg/kg/d × 2d	200 mg/kg/d × 2d	No	No	No	200 mg/kg/d × 2d	No	200 mg/kg/d × 2d
G-CSF	Yes	Yes	Yes	No	Yes	No	No	No	No
Blood transfusion	Platelet transfusion	No	Platelets, red blood cells	No	Platelet transfusion	Platelets, red blood cells	No	No	red blood cells
Symptom relief	The temperature improved after 1 day of medication, and the respiratory symptoms completely improved after 5 days	Body temperature improved after 2 days of medication.	The temperature improved after 1 day of medication	After 4 days of medication, the body temperature improved, and cough occasionally	After 3 days of medication, the body temperature gradually improved	Symptoms took a turn for the better after 5 days of medication.	The temperature improved after 2 days of medication, and the respiratory symptoms completely improved after 4 days	After 3 days of medication, the temperature improved, and after 5 days, the respiratory symptoms were completely improved	After 1 day of medication, the body temperature improved and cough was observed for 1 week
Virus clearance time	5 days	15 days	5 days	6 days	5 days	5 days	5 days	5 days	7 days
New occurrences after medication	Hypertension (HBP 136/86mmHg)	No	No	No	No	No	No	No	No
Fever/infection recurred within 2 weeks	No	No	Yes	No	Yes	No	No	No	No

### Non-Paxlovid-treated control group

2 cases (18.2%) were inoculated with the new coronavirus vaccine, 10 cases (90.9%) had a fever, 7 cases (63.6%) had respiratory symptoms, 2 case (18.2%) had digestive tract symptoms, and 2 case had neurological symptoms (18.2%), no skin and mucous membrane damage, muscle pain, conjunctivitis, etc. In the early stage of infection, there was 5 case (45.5%) with abnormal white blood cells, 4 cases (36.4%) with anemia, 3 cases (27.3%) with thrombocytopenia, 0 cases with elevated CRP, and 0 cases with elevated PCT. None of the 11children received antiviral or antibacterial drug treatment. The average duration of fever was 0–3 days (mean 1.91 ± 0.944 days), the clinical symptoms improved within 2–14 days, and the virus clearance took 5–27 days. Three days after the body temperature improved, there were 3 cases of fever again, all of which were considered secondary bacterial infections after the new coronavirus infection (shown in Table 4).

**Table 4:** Clinical data of 11 children with hematological diseases in group B infected with novel coronavirus.

Patients	10	11	12	13	14	15	16	17	18	19	20
Gender	Female	Female	Male	Male	Male	Female	Male	Male	Male	Female	Male
Age(years)	6	5	8	11	6	3	11	4	5	2	2
Weight (kg)	18	20	27	32	27.5	16	30.5	16	20.5	10.5	14
Basic illness	ALL (B, IR, CR)	ALL (T, IR)	ALL (B, HR)	PRCA	AA (Erythroid and megakaryocytic lineages)	ALL (B, LR)	ALL (B, IR)	ALL (B, IR)	ALL (B, IR)	ALL (B, LR)	ALL (B, LR)
COVID-19 Vaccination Status	0	2	0	0	2	0	0	0	0	0	0
Fever	Yes, 38.4°C	No	Yes, 39°C	Yes, 39°C	Yes, 38°C	Yes, 38.9°C	Yes, 39°C	Yes, 38.5°C	Yes, 39°C	Yes, 38.6°C	Yes, 38.5°C
Duration of fever	1 day	0 day	2 days	3 days	2 days	2days	1day	3days	3days	2days	2days
cough/stuffy nose/runny nose/sore throat	No	Cough、sore throat	Cough、runny nose	Cough/stuffy nose/runny nose/sore throat	Cough	No	Cough	No	Cough	Cough	No
Rash/mouth sores	No	No	No	No	No	No	No	No	No	No	No
Conjunctivitis	No	No	No	No	No	No	No	No	No	No	No
Headache/dizziness	No	Dizziness	No	No	No	No	Headache	No	No	No	No
Muscle pain	No	No	No	No	No	No	No	No	Yes	No	No
Vomiting/diarrhea/stomachache	Stomachache	No	No	No	No	No	Vomiting	No	No	No	No
The first new coronavirus nucleic acid CT value	ORF/N 28.23/28.84	ORF/N 29.35/29.64	ORF/N 28.63/28.23	ORF/N 32.64/32.29	ORF/N 32.10/32.47	ORF/N 26.35/26.02	ORF/N 23.12/23.07	ORF/N 28.63/28.23	ORF/N 32.64/32.29	ORF/N 32.64/32.29	ORF/N 32.10/32.47
WBC( × 10^9^/L)	6.78	16.34	4.97	9.48	4.69	3.43	3.73	3.95	4.75	2.89	5.72
ANCA( × 10^9^/L)	4.57	11.61	2.61	6.61	1.39	1.86	1.15	1.39	1.95	1.28	5.06
LC( × 10^9^/L)	1.19	1.62	1.94	2.25	3.11	1.15	2.16	1.71	2.51	1.37	0.51
Hb(g/L)	143	87	95	70	70	112	110	114	137	119	113
PLT( × 10^9^/L)	121	92	204	362	22	338	94	328	294	171	419
CRP (mg/L)	1.23	0.86	0.92	0.75	0.90	0.23	0.43	0.38	1.68	0.21	0.45
PCT (ng/ml)	<0.05	<0.05	<0.05	<0.05	<0.05	<0.05	<0.05	<0.05	<0.05	<0.05	<0.05
Relief time	Fever improved after 1 day	1 week	No	No	Fever improved after 2 days	2days	14days	3days	7days	4days	2days
Virus Clearance Time(day)	10	5	27	22	12	9days	14days	15days	10days	5days	9days
Recurrence of fever/infection within 2 weeks	No	Yes	Yes	Yes	No	No	No	No	No	No	No

**PRCA**, pure red cell aplasia.

### Comparison of paxlovid-treated and non-treated groups

Clinical symptoms were similar in both AB groups The children in group A took paxlovid within 5 days after the onset of symptoms, and the fever lasted for 1–6 days, clinical symptoms resolve in 1–30 days. While the children in group B had a fever for 0–3 days, and the clinical symptoms resolved within 2–14 days. The virus clearance time in group A was shorter than in group B,. The inflammatory indexes CRP and PCT in group A were significantly higher than those in group B, (*P *< 0.05) (shown in Table 5).

**Table 5:** Comparison of clinical manifestations and laboratory test results of two groups infected with novel coronavirus.

Variables	Group A (*n* = 9)	Group B (*n* = 11)	*Fisher* test	*P* value
Vaccination Status	2	2	0.158	0.700
Duration of Fever(day)	2.89 ± 1.965	1.91 ± 0.944	11.569	0.008
Respiratory Symptoms	6	7	2.761	0.131
Vomit	1	1	2.037	0.154
Stomachache	1	1	0.020	0.887
Complicated with organ damage	0	0	–	–
CRP	15.60 ± 6.948	0.73 ± 0.452	15.139	0.004
PCT	0.447 ± 0.363	<0.05	50.837	<0.001
Combined Antibiotics	9 (100%)	0	11	0.001
Virus Vlearance time(day)	6.44 ± 3.283	15.20 ± 9.039	6.323	0.033
Fever again within 2 weeks	2	3	0.782	0.376

### Results of follow-up

Group A: Case 3 had increased sleep for 1 month; Case 4 had recurrent cough for 1 month after discharge. Case 5 was re-hospitalized due to gingivitis and sepsis, but no respiratory symptoms recurred.

Group B: Patient 16 had loss of appetite, and patient 19 had easy fatigue, which could be improved by increasing mild aerobic exercise.

## Discussion

Children infected with the novel coronavirus rarely progress to severe pneumonia, and their symptoms are milder than adults (17). This may be due to children's stronger innate immunity, which can better clear the virus, while adaptive immunity is weaker, and the inflammatory immune response is milder and other factors (18). Children with hematological tumors or after hematopoietic stem cell transplantation and receiving large doses of chemotherapy drugs and hormones have low immunity, decreased ability to clear respiratory secretions, and significantly increased risk of upper respiratory tract and lung infections (19–21). A study of 131 children with tumors and hematopoietic stem cell transplantation infected with the novel coronavirus proved that comorbidities, other infections, and neutrophil deficiency were significantly associated with increased disease severity (21). Other studies have shown that vaccination is the most valuable and effective strategy for preventing severe COVID-19 among children with underlying diseases (22, 23). Only 20% of the children in this study were vaccinated, and the rest had not been vaccinated due to vaccination contraindications such as infection and chemotherapy. There was no difference in the vaccination status between group A and group B. In this study, 20 children with hematological neoplasms infected with the novel coronavirus Omicron variant The clinical symptoms of 20 children infected with the new coronavirus were similar, manifested as fever, respiratory, and gastrointestinal symptoms. None of them had severe pneumonia or secondary organ insufficiency.

The safety and effectiveness of Paxlovid in adult COVID-19 patients have been proven (8, 24). According to research in the United States during the Omicron epidemic period from April to August 2022, Paxlovid reduced the risk of hospitalization after COVID-19 infection by up to 51% (25), However, currently, Paxlovid is approved by the US FDA for people over the age of 12^9^. In this study, 9 children with hematological diseases in group A had 2 or more high-risk factors for severe and critical illness, and the median age was 6. After consulting with the Department of Infectious Diseases and the Department of Pharmacy and obtaining the signed consent of the family members, they were actively given anti-coronavirus treatment, with sound curative effects and no noticeable adverse reactions.

In this study, 20 children were divided into two groups according to whether they received Paxlovid treatment or not, and the results showed that the viral clearance time was shorter in group A than in group B. The mean duration of fever and the mean duration of clinical symptom relief were close in the two groups AB, while the inflammatory indexes CRP and PCT were significantly higher in 66.7% of children in group A, suggesting the possibility of co-infection with bacterial infection, which in turn affected the effect of Paxlovid drug on The observation of the improvement of clinical symptoms in children with neocoronavirus infection. In addition, fever reappeared in 25% of the cases in this study after 3 days of temperature improvement, suggesting that children with hematologic underlying diseases, especially after transplantation or long-term chemotherapy, should pay attention to the signs of bacterial infection after SARS-CoV-2 infection along with antiviral treatment and timely combination of antibacterial drugs if necessary.

It has been reported that the clearance time of the SARS-CoV-2 virus in the general population is 5–10 days, but people with compromised immune function may take longer to clear the virus (26). For example, the median time to clear SARS-CoV-2 in children after allogeneic hematopoietic stem cell transplantation is 20–27 days (27, 28). The virus clearance time of Case 1 in this study was 5 days, which is similar to other research results (29, 30). Paxlovid treatment is beneficial to shorten the course of the disease and avoid delaying the treatment of the primary disease. However, the functions of various organs in children are immature, especially those with primary diseases of hematological tumors. During the treatment period, it is necessary to pay attention to the interaction between drugs (31).

The liver in the human body mainly metabolizes cyclosporin, dasatinib, and other drugs, and CYP3A4 is an essential catalytic enzyme for drug metabolism (32). Paxlovid is a compound preparation of Naimatevir and Ritonavir. Ritonavir is used for HIV treatment and is a highly effective antiviral drug synergist in the anti-new crown treatment (33). Since Ritonavir has vigorous CYP3A4 inhibitory activity, it can significantly affect the metabolism of other drugs in the liver, so Paxlovid interacts with other drugs.

According to the observation of the clinical symptoms of 9 children after taking Paxlovid, 2 cases had the symptoms of bitter taste, which improved spontaneously after a few days. According to the time axis, it may be caused by the drug. In case 1, within 3 months after hematopoietic stem cell transplantation for thalassemia, cyclosporine A immunosuppressive therapy was essential, while oral prednisone was administered to prevent GVHD, and hypertension developed after Paxlovid antiviral therapy. A pharmacokinetic study showed that the combined use of cyclosporine and ritonavir (100 mg) increased the total exposure of cyclosporine by 5.8-fold3 (34). Hypertension is a common adverse reaction of cyclosporine, which is positively correlated with the plasma concentration of cyclosporine (35). The National Institutes of Health COVID-19 treatment guidelines recommend adjusting the dose of cyclosporine during nematrevir/ritonavir administration but do not provide specific dosage recommendations (36). A study of solid organ transplantation and HIV-infected patients recommended an 80 percent reduction in the dose of cyclosporine when using adult doses of ritonavir (37). In this study, case 1 weighed less than 20 kg, received ritonavir 50 mg, and reduced the dose of cyclosporine by 66.6%, but still had high blood pressure, and the resting blood pressure was 136/86mmHg (more than 99% of the blood pressure of children of the same age, sex, and height).

## Conclusion

In conclusion, paxlovid is recommended for children with hematologic underlying immunosuppression or pneumonia infected with 2019-nCoV before severe disease develops. It can shorten the virus clearance time and reduce the risk of developing severe disease. However, at the same time, it is necessary to pay attention to whether there are signs of secondary bacterial infection, and it is necessary to combine antibiotics in time for treatment. According to this study, there is no apparent adverse reaction when the drug is used in children aged 12 and under. Because the liver's metabolic function in children is different from that in adults, further research is needed on the effect of drugs on interactions. The priority between these two should be considered when carrying out anti-coronavirus and treating other diseases. In addition, in this study, children with primary hematological diseases without immunosuppression had mild symptoms and short self-healing time. Due to the lack of safety and pharmacokinetic studies, paxlovid is not recommended for the time being. In the future, more research and data are needed to elaborate further and demonstrate the application of paxlovid in children with blood diseases who are co-infected with the new coronavirus.

## Data Availability

The original contributions presented in the study are included in the article/Supplementary Material, further inquiries can be directed to the corresponding author/s.
